# The Investigation of the Silica-Reinforced Rubber Polymers with the Methoxy Type Silane Coupling Agents

**DOI:** 10.3390/polym12123058

**Published:** 2020-12-20

**Authors:** Sang Yoon Lee, Jung Soo Kim, Seung Ho Lim, Seong Hyun Jang, Dong Hyun Kim, No-Hyung Park, Jae Woong Jung, Jun Choi

**Affiliations:** 1 Human Convergence Technology R&D Department, Korea Institute of Industrial Technology (KITECH), Ansan 15588, Korea; sangyoon@kitech.re.kr (S.Y.L.); kimjungsoo11@kitech.re.kr (J.S.K.); tmdgh2603@kitech.re.kr (S.H.L.); seonghyun@kitech.re.kr (S.H.J.); dhkim@kitech.re.kr (D.H.K.); nohyung@kitech.re.kr (N.-H.P.); 2 Department of Advanced Materials Engineering for Information & Electronics, Kyung Hee University, Yongin 17104, Korea

**Keywords:** rubber, silane coupling agent, masterbatch, silica filler, composite, tire tread

## Abstract

The methoxy-type silane coupling agents were synthesized via the modification of the hydrolyzable group and characterized to investigate the change in properties of silica/rubber composites based on the different silane coupling agent structures and the masterbatch fabrication methods. The prepared methoxy-type silane coupling agents exhibited higher reactivity towards hydrolysis compared to the conventional ethoxy-type one which led to the superior silanization to the silica filler surface modified for the reinforcement of styrene-butadiene rubber. The silica/rubber composites based on these methoxy-type silane coupling agents had the characteristics of more developed vulcanization and mechanical properties when fabricated as masterbatch products for tread materials of automobile tire surfaces. In particular, the dimethoxy-type silane coupling agent showed more enhanced rubber composite properties than the trimethoxy-type one, and the environmentally friendly wet masterbatch fabrication process was successfully optimized. The reactivity of the synthesized silane coupling agents toward hydrolysis was investigated by FITR spectroscopic analysis, and the mechanical properties of the prepared silica-reinforced rubber polymers were characterized using a moving die rheometer and a universal testing machine.

## 1. Introduction

**Figure 1:**
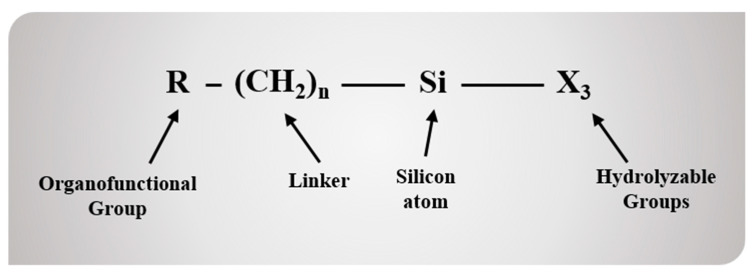
The chemical structure of the conventional silane coupling agents (SCA)s.

Rubber compounds, a key material in the automotive tire industry, are manufactured by mixing natural or synthetic rubber with organic/inorganic fillers and are often used as tread materials for automobile tire surfaces. Styrene-butadiene rubber (SBR) is a representative synthetic rubber that is applied to a wide range of products, such as automotive tire treads, rubber belts, and shoes, among which the automotive tire tread sector accounts for more than 70% of the SBR market [1,2,3]. A certain amount of rigid fillers must be added in the rubber matrix for property improvement or cost reduction [4,5]. Carbon black (CB) and silica are widely used as fillers in rubber compounds [6,7,8]. The required properties of the rubber compounds used as tire tread materials are largely classified into three categories: (1) rolling resistance, which is related to fuel efficiency, (2) wet traction, which is relevant to directional stability, and (3) abrasion resistance, which is associated with tire lifetime. The required physical properties are affected by various factors and are in a trade-off relationship with each other, and thus, it is almost impossible to improve all properties simultaneously. The most important factor that affects the properties of rubber compounds is the interaction between the filler and the rubber, which is known to influence the three properties that are essential for rubber compounds. The use of silica has recently increased for improving the rolling resistance and wet traction properties of rubber compounds instead of CB. However, abrasion resistance, mechanical properties, and processing characteristics are required to be improved [9,10]. In order to address this issue, the method for containing higher silica content in rubber compounds was suggested in this study. The rubber compounds with high silica content have shown improvements in the mechanical properties and abrasion resistance with increasing filler content. Owing to the presence of hydrophilic components such as silanol groups on its surface, silica has a low affinity with the hydrophobic rubber, resulting in drawbacks such as poor filler dispersion within the rubber compound and poor filler–rubber interaction [11,12]. To address such problems, silane coupling agents (SCAs) have been employed in numerous studies [13,14]. SCAs with different chemical structures have been commercially developed for surface modification of silica [15,16]. The structure of the conventional SCAs is shown in Figure 1, and it consists of a hydrolyzable group that bonds with silica via hydrolysis, a silicon atom, a linker, and an organofunctional group that vulcanizes or exhibits miscibility with rubber. When using 3,3’-bis(triethoxysilylpropyl)disulfide (TESPD), the most widely used commercial SCA, to compound the SBR and the filler with high uniformity, significant problems occurred such as limitations in the reactivity towards hydrolysis, side reactions related with the aggregation of the SCA owing to its three alkoxy groups, and the ethanol by-product from the introduction of silica which weakens the bonding force between the SBR and the silica surface [17]. The newly synthesized methoxy-type SCAs in this study settled those forementioned problems.

In recent years, there has been a growing interest in the wet masterbatch (WMB) technology, a new mixing technique to improve the processability and dispersion of silica for enhanced abrasion resistance, mechanical properties, and dynamic viscosity. WMB is a solidification technique, which involves dispersing fillers in a SBR latex solution, where an SCA hydrolyzed in the liquid state reacts with silica to create a surface-modified silica slurry, which is then mixed with the latex solution to undergo the manufacturing process for producing a masterbatch [17,18]. WMB has the advantages of being able to accommodate a large amount of fillers while achieving a higher filler dispersion within the rubber matrix and a good filler–rubber interaction. Mun et al. functionalized an emulsion SBR (ESBR) structure and prepared a silica-reinforced rubber compound through the WMB process. Then, they compared the physical properties of the silica-reinforced rubber with those obtained by the dry masterbatch (DMB) method [19]. Unlike the previous study, our study focused on the modified SCA structure and proved its superiority by comparing the physical properties of the silica-reinforced rubber compound produced through the WMB and DMB methods. In particular, this water-based WMB technique does not produce toxic methanol during the compounding process, and thus, the methoxy-type SCAs can be applied without the environmental concerns. However, this technique requires significant research, as there is little knowledge about aspects such as the pH of the silica slurry, type of aggregate, and drying conditions. This technique has more complex manufacturing and SCA hydrolysis conditions than the conventional DMB technique.

In this study, a novel methoxy-type SCA structure was synthesized via the modification of the hydrolyzable group and applied to rubber compounds to allow maximizing the silica–SBR interaction, preventing SCA aggregation and increasing the solubility in water. To produce a silica–SBR compound, the optimized WMB technique was applied as well as the conventional DMB technique, and the reactivity of the synthesized SCAs towards hydrolysis, the loss ratio of silica, mechanical properties, abrasion resistance, and processing characteristics of the rubber compound were compared and investigated.

## 2. Materials and Methods

### 2.1. Materials

(3-mercaptopropyl)methyldimethoxysilane (MPDMS, ≥95%), 2,2’-dithiodipyridine, dichloromethane (anhydrous, 99.8%), petroleum ether, and isopropyl alcohol (IPA, 99.7%) were purchased from Sigma-Aldrich, St. Louis, MO, USA. SBR 1723 (styrene content: 23.5%, oil type: TDEA 37.5 phr, mooney viscosity (ML1 + 4): 49, coagulant: acid salt) was obtained from Kumho Petrochemical, Seoul, Korea. F-175 silica cake (pH: 7.0, loss on drying/105 °C × 2H: 6.0%, ignition loss at 1000 °C: 13.0%, oil absorption: 250 mL/100 g, Brunaeur–Emmett–Teller (BET): 175 m^2^/g) was purchased from Namhae Chemical, Yeosu, Korea. Bis[3-(triethoxysilyl)propyl] disulfide (TESPD (Si-75)) was purchased from Evonik Industries, Essen, Germany. Sodium hydroxide (NaOH, ≥96%) was obtained Samchun, Seoul, Korea. Various compounding processing additives such as zinc oxide (ZnO), stearic acid (S/A), and N-(1,3-dimethyl-butyl)-N’-phenyl-p-phenylenediamine (6PPD) were purchased from Sigma-Aldrich, USA. In the final compounding step, sulfur (Samchun, Seoul, Korea) was used as a crosslinking agent. N-cyclohexyl-2-benzothiazole sulfonamide (CBS) (Tokyo Chemical Industry, Tokyo, Japan) and diphenyl guanidine (DPG, Sigma-Aldrich, St. Louis, MO, USA) were used as crosslinking accelerators.

### 2.2. Synthesis of SCAs

**Figure 2:**
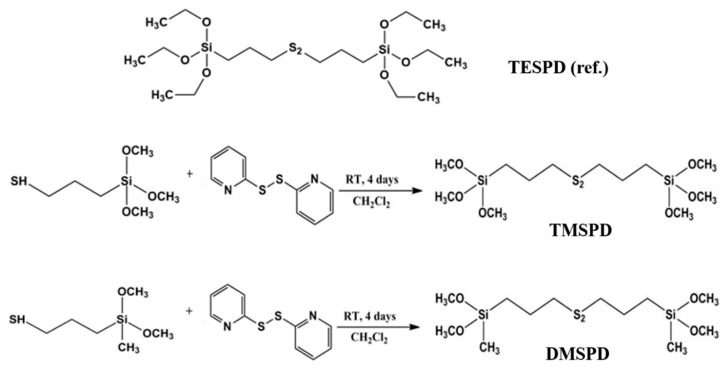
The chemical structure of the prepared SCAs.

The chemical structure of the methoxy SCAs developed in this study and its simplified synthesis method are shown in Figure 2.

#### 2.2.1. Synthesis of 3,3’-Bis(trimethoxysilylpropyl) Disulfide (TMSPD)

First, 20 mL of dichloromethane and 2 g of 2,2’-dithiodipyridine were mixed in a three-neck round-bottom flask connected to a reflux condenser and nitrogen. Then, 4.45 g of excess (3-mercaptopropyl)trimethoxysilane was quickly added to the previously prepared solution using a syringe, and the mixture was allowed to react at room temperature for 4 days. The reaction product was mixed with petroleum ether and distilled water and then repeatedly extracted for three times. The extracted material was distilled under reduced pressure to remove the solvent and dried in a vacuum oven at 60 °C.

^1^H NMR (500 MHz, CD_2_Cl_2_): δ (ppm) 3.54 (s, 18 H), 2.70 (m, J. = 11.4 Hz, 4 H), 1.77 (m, J. = 13.2 Hz, 4 H), 0.76 (m, J. = 11.2 Hz, 4 H). HRMS (m/z): calcd. for (C_12_H_30_O_6_S_2_Si_2_): 390.10.

#### 2.2.2. Synthesis of 3,3’-Bis(dimethoxymethylsilylpropyl) Disulfide (DMSPD)

A total of 20 mL of dichloromethane and 2 g of 2,2’-dithiodipyridine were stirred in a three-neck round-bottom flask connected to a reflux condenser and nitrogen. Then, 4.45 g of excess MPDMS was quickly added to the earlier solution with a syringe and reacted at room temperature for 4 days. Once the reaction was completed, the product was mixed with petroleum ether and distilled water and extracted repeatedly for 3 times. After removing the solvent via distillation under reduced pressure, the product was dried in a vacuum oven at 60 °C.

^1^H NMR (500 MHz, CD_2_Cl_2_): δ (ppm) 3.48 (s, 12 H), 2.69 (m, J. = 10.8 Hz, 4 H), 1.74 (m, J. = 13.4 Hz, 4 H), 0.69 (m, J. = 12.2 Hz, 4 H), 0.10 (s, 6 H). HRMS (m/z): calcd. for (C_12_H_30_O_4_S_2_Si_2_): 358.22.

### 2.3. Preparation of Silica/SBR Composites

#### 2.3.1. Preparation of the Materials

SBR 1723 latex was used as the rubber matrix component, and the F-175 silica cake, the commonly used silica in the current tire industry with a specific surface area of 175 m^2^/g, was used as the reinforcing filler component to create tread rubber compounds. A silica cake was used as the form of filler for the WMB composite. The silica cake referred to the silica dispersed in an aqueous solution, where the silica preparation only proceeded to the sedimentation stage, while silica powder referred to the dried state of the silica cake. Silica powder pressed into a granule form was used as the filler for the DMB composite.

#### 2.3.2. Preparation of WMB Silica/SBR Composites

The WMB manufacturing process first requires modification of the silica surface using an SCA, where the hydrolysis of the alkoxy groups of the SCA and their conversion to silanol groups is essential for introducing the SCA to the silica surface. To hydrolyze the alkoxy groups of the prepared SCA, 13.14 g of SCA and 25.0 g of IPA were vigorously mixed in a vessel at room temperature as 75 g of H_2_O was slowly added, and acetic acid was added to the mixture until the pH reached 4 [17,18]. After allowing the reaction to proceed for an additional hour, the produced silane hydrolysates were added to 164.22 g of silica cake and mixed after adding 465.76 g of distilled water. A 25 wt % NaOH solution was added to the mixture until the pH reached 8, and the solution was stirred at 70 °C for 4 h and then dried to prepare the surface-modified silica [20,21]. It is necessary that the SCA dissolves well in an aqueous solution to hydrolyze the alkoxy groups of the SCA in the process solvent, and thus, the WMB manufacturing method was unable to produce a proper, uniform sample with TESPD due to its poor dissolution in water. After that, 87.98 g of the treated distillate aromatic extracted oil emulsion was mixed with 234.6 g of SBR 1723 latex to produce an oil-extended SBR latex. The silica surface-treated with SCA was mixed with 1 L of distilled water to produce a surface-modified silica dispersion solution. Then, the oil extended SBR latex and the surface-modified silica dispersion solution were mixed. After mixing was completed, sulfuric acid was added, until the pH reached 4 to solidify the mixture, which was then washed three times with distilled water and dried in a convection oven for 6 h. Next, the mixing compounds were prepared according to the formulation in Table 1, and the detailed procedure was explained in the following chapter. By using the WMB method, a much higher production efficiency was achieved under a mixing temperature of 80 °C and a mixing time of 6 min.

#### 2.3.3. Preparation of DMB Silica/SBR Composites

In the DMB manufacturing process, the SCA was introduced during the compounding process along with the silica, which was not surface-modified in advance in order to achieve direct interaction with the rubber through the silanization reaction. However, due to limitations in the silanization reaction condition (>150 °C) that resulted in high temperature settings in a mixer and increased mixing times during compounding, the process suffered from low processing efficiency and difficulty in removing alcohol by-products from the silanization reaction inside the kneader. Next, the mixing compounds were prepared according to the formulation in Table 1 using a kneader, which was a closed-type mixer. The mixing conditions were set to 75% of the internal capacity of the mixer as an appropriate filler loading, and in the case of the DMB, the internal mixing temperature was set to maintain 150 °C. In addition, upon setting the rotor speed of the kneader to 25 rpm, the materials were added in the order of rubber, filler, and other processing aids, which were all mixed for a total time of 12 min. In the second stage of the mixing process (the finish masterbatch, FMB), which was the final compounding stage after the first mixing stage, sulfur and an accelerator were added in an 8-inch two-roll mill (rotor speed ratio, 1:1.4) and mixed in the kneader at an internal mixing temperature of 50 °C and a mixing time of 2 min to increase the dispersion efficiency of a crosslinking agent and an accelerator. Following the kneader mixing process, the rubber compounds were formed into sheets with a thickness of 2.4 mm in the two-roll mill and subsequently cooled for 12 h.

### 2.4. Measurement

#### 2.4.1. FTIR Spectroscopic Analysis

An FTIR-620 (JASCO, Tokyo, Japan) was used, and the attenuated total reflectance technique was utilized to obtain the IR spectra. The samples for the FTIR measurement were prepared by making a pallet with KBr. After sampling the raw materials, an appropriate amount was placed on the IR prism and was secured with a micrometer, after which a 4000–500 cm^−1^ spectral range was measured.

#### 2.4.2. Gel Permeation Chromatography (GPC)

The analytes were dissolved in tetrahydrofuran, and the sample solution was injected into the column. The flow rate of the sample solution was set to 1 mL/min for 60 min, and a pulse damper was installed to minimize pump pulsation.

#### 2.4.3. Thermogravimetric Analysis (TGA)

The silica contents of the WMB samples were measured. In this experiment, the WMB samples contained SBR and silica, an inorganic material, and thus, the SBR underwent weight reduction via thermal decomposition while silica remained in the form of ash, which allowed for the measurement of silica content. The temperature was initially maintained at 25 °C for 5 min and raised to 900 °C at a rate of 10 °C/min. Then, the temperature was maintained at 900 °C for 5 min, and the remaining ash was weighed to determine the silica content.

#### 2.4.4. Deutsches Institut für Normung (DIN) Abrasion Test

Abrasion tests were performed in accordance with ASTM 53,516, where a DIN sample mold was used to vulcanize a cylindrical sample with a diameter of 16 mm and a height of 8 mm. The rubber specimen for the DIN abrasion test was subjected to frictional wear using an abrasive sheet attached to an abrasion tester to complete an abrasion distance of 40 m at a drum rotation speed of 40 rpm, and the abrasion resistance of the test specimen was determined by measuring the mass loss.

#### 2.4.5. Mechanical Properties

In accordance with ASTM D412, a dumbbell-shaped specimen was prepared to measure the mechanical properties of the vulcanized rubber. Measurements were conducted using a universal testing machine (UTM, KSU-05M-C, Ansan, Korea) with a 500 N load cell and a crosshead speed of 500 mm/min. The measured results were presented as 100% modulus, 300% modulus, tensile strength, and percent elongation.

## 3. Results and Discussion

**Figure 3:**
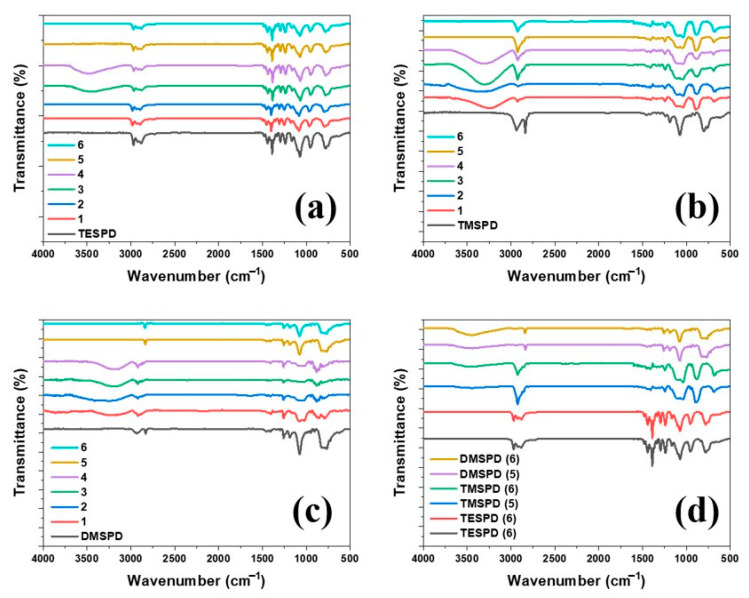
FTIR spectra for 1 h hydrolysis reactions of the SCAs: (**a**) TESPD; (**b**) TMSPD; (**c**) DMSPD; and (**d**) spontaneous reactions of samples 5 and 6 during 10 days.

To evaluate the reactivity towards hydrolysis, which is the most important feature of the SCA, hydrolysis reactions under various conditions were carried out, as presented in Table 2. The samples for the FTIR measurement were prepared by making a pallet with KBr. As shown in Figure 3, the results demonstrated that TMSPD and DMSPD showed higher reactivity in various compositions of co-solvent compared to TESPD due to their much higher solubility in water. Owing to the presence of ethoxy groups, TESPD showed low reactivity towards hydrolysis, and the silanol group (Si–OH) peak at 3426 cm^−1^ was not observed, except under conditions 3 and 4 where IPA was a major component, whereas TMSPD and DMSPD contained methoxy groups that allowed all hydrolysis reactions to occur easily within 1 h, except under conditions 5 and 6 where no acetic acid was added. In addition, as presented in Figure 3d, TMSPD and DMSPD spontaneously hydrolyzed even under conditions where no acid catalyst was added when they were maintained in a process solvent for more than 10 days. However, they also exhibited superior storage stability of more than 6 months under typical room temperature and dry conditions. While TESPD required more than 78% of IPA as a process solvent because of its low water solubility, TMSPD and DMSPD readily hydrolyzed in the process solvent even when the water content was increased to 96%, and thus, they showed economic feasibility and environmental friendliness [1,7,18]. They can also be applied to the water-based WMB method, which is known to have a high processing efficiency for manufacturing mixing compounds in the tire industry.

**Figure 4:**
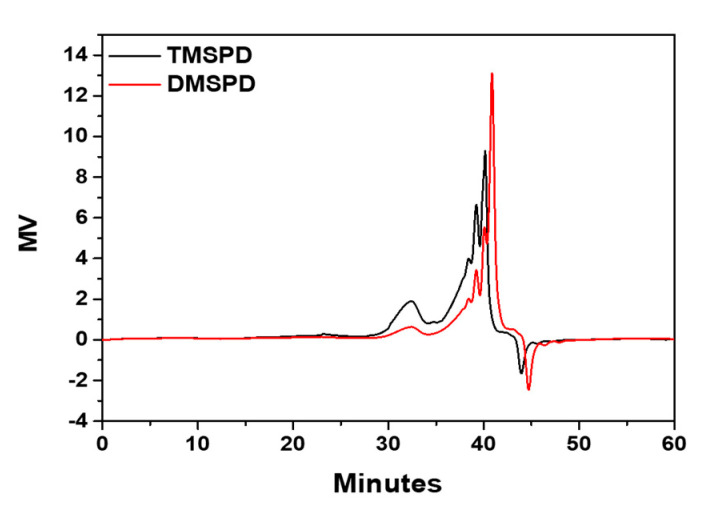
Gel permeation chromatography (GPC) data for methoxy-type SCAs after condensation side reactions.

In general, alkoxy-based SCAs generate alcohol after the hydrolysis reaction, which is a by-product that not only hardens by aggregation through a condensation reaction but also has an adverse effect on the mechanical properties of the final rubber composites. To investigate the tendency of methoxy-based SCAs towards the side reactions, the spontaneously hydrolyzed samples from the previous experiment reacted in a 5% NaOH aqueous solution at 40 °C for 4 h. The GPC analysis was carried out after the reaction to determine the condensation products of high molecular weight, and the obtained results, shown Figure 4, revealed that DMSPD had a lower tendency towards a condensation side reaction compared to TMSPD since it showed a smaller ratio of high molecular weight components. It was speculated that the probability of a side reaction decreased because DMSPD contains two methoxy groups, fewer than those in TMSPD. Thus, DMSPD was considered to have a better storage stability than TMSPD.

**Figure 5:**
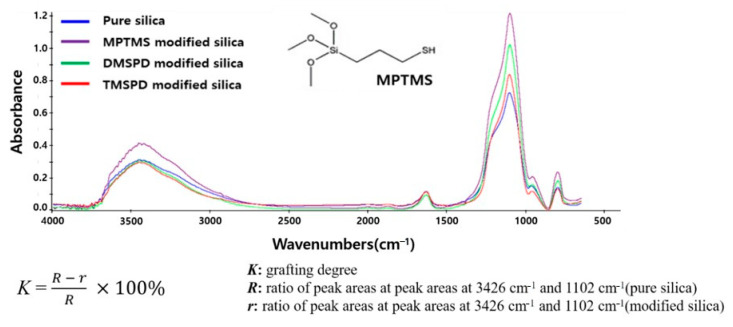
FTIR spectra of the modified silica cake with the prepared SCAs.

For a comparative evaluation of the extent to which silica became hydrophobic based on the SCA type, the FTIR spectrum of the modified silica cake in the Figure 5 was analyzed to measure the grafting degree (K). The grafting degree (K) was obtained using the equation shown below, and MPTMS, which is known to easily hydrolyze, was used as the reference SCA, as the TESPD does not undergo the hydrolyzation easily. The peak at 3426 cm^−1^ in the graph represents the silanol group (Si–OH), and the peak at 1102 cm^−1^ corresponded to the Si–O–Si group. As silica modification proceeded, the value of the grafting degree increased, because the presence of the silanol group (Si–OH) of the prepared silica cake diminished and the peak area at 3426 cm^−1^ was relatively reduced. As can be observed in the K values compared in the Table 3, TMSPD and DMSPD showed a higher grafting degree than MPTMS and ultimately had an excellent ability to form Si–O–Si bonds with silica, and thus, they were considered to be more advantageous for silica to become hydrophobic. DMSPD, which has a di-methoxy group, was even more favorable for making the silica hydrophobic despite its fewer hydrolyzable groups. This result was mainly because it showed an enhanced silanization reaction due to its lower steric hindrance than the tri-methoxy silane and the suppressed side reaction after being introduced to the silica surface. The detailed discussions are provided in the following paragraphs.

**Figure 6:**
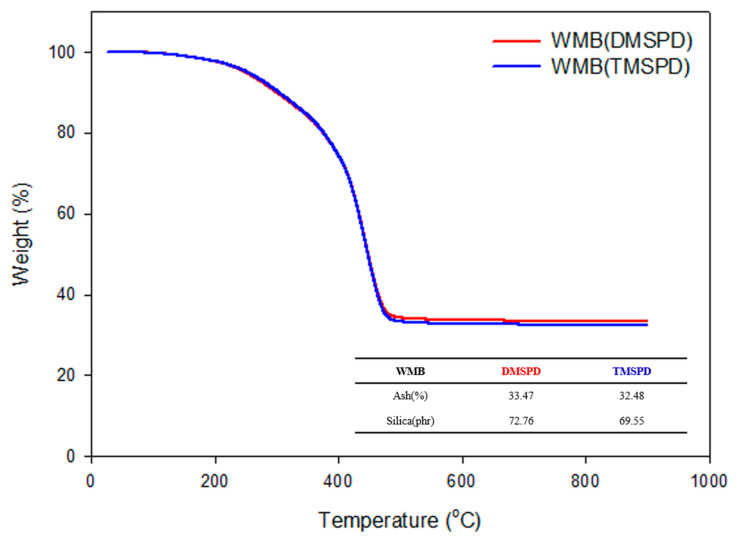
The evaluation of remaining silica content in the wet masterbatch (WMB) sample with methoxy-type SCAs.

The evaluation of the remaining silica content in the WMB sample was performed using TGA, where the sample was maintained at 25 °C for 5 min and then raised to 900 °C at a 10 °C/min ramp. After maintaining the temperature at 900 °C for 5 min and measuring the remaining ash content to determine the silica content, DMSPD showed a lower loss of silica compared to TMSPD as indicated in Figure 6. The prepared silica fillers were more efficiently modified and introduced to the rubber composites when they were silanized with the DMSPD SCA. In other words, similar to the previous result, DMSPD with the di-methoxy group was more advantageous for making silica hydrophobic compared to TMSPD.

**Figure 7:**
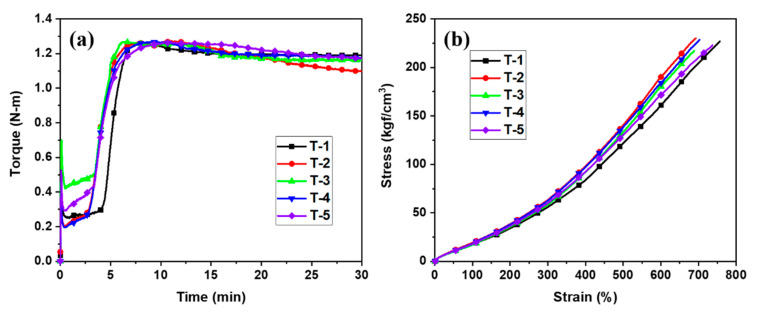
(**a**) The moving die rheometer (MDR) data for dry masterbatch (DMB) and WMB with SCAs. (**b**) The stress–strain curve of samples T-1–T-5.

The final compound mixed to completion in the second mixing stage mentioned earlier was characterized using a moving die rheometer (MDR) to measure the torque of an uncured compound at 160 °C for 30 min under an oscillation angle of ±1°, as shown in Figure 7a. The measured torque value was used to determine the maximum torque (T_max_), minimum torque (T_min_), and optimum cure time (t_90_) in Table 4. The vulcanization of the rubber specimen was conducted in a hot process over an optimum cure time (t_90_ + 2 min) [22]. The result comparison between the samples showed that the methoxy-type SCAs rather than the ethoxy-type SCAs were more favorable towards processability (e.g., Mooney viscosity) and cure rate. This outcome seemed to arise from the fact that methoxy groups exhibit a higher reactivity towards hydrolysis and have a lower steric hindrance allowing the formation of compact bonds with silica, and thus, the silanization reaction occurs to a greater extent [23,24]. As a result, the effect of surface modification on silica became greater and the hydrophobicity of silica increased, leading to suppressed reagglomeration and reduced scorch generation. Moreover, in the comparison between the methoxy-type SCAs, fewer agglomerates were produced, Mooney viscosity was reduced, and the improvement (T_max_–T_min_) in the vulcanization characteristics was greater in DMSPD than in TMSPD, because the former has fewer lateral methoxy groups than the latter, and therefore, the hydrophilicity of the modified silica was weaker [25]. The WMB samples had a higher value of the difference between the maximum and minimum torques (T_max_ – T_min)_ than the DMB samples, which demonstrated the property enhancement due to curing was considered to be greater. Moreover, the WMB samples had a lower Mooney viscosity than the DMB samples and demonstrated superior processability. The cure rate of DMSPD was higher than that of TMSPD in both DMB and WMB samples.

**Figure 8:**
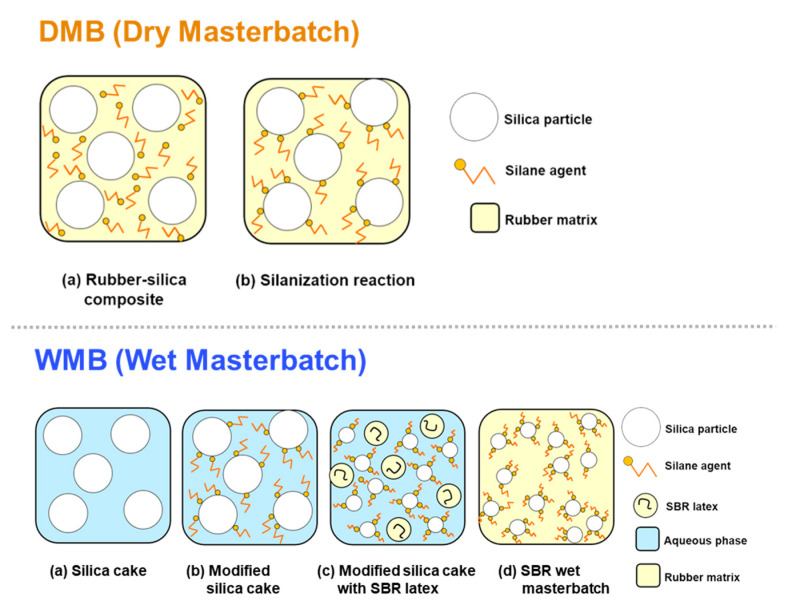
The comparison of the DMB and WMB fabrication processes.

Figure 7b shows the stress–strain curves of the DMB and WMB samples with the prepared SCAs. The abrasion resistance was measured under identical conditions conforming to the DIN 53,516 measurement procedure, and the mass losses before and after investigation were measured. The mechanical properties were also measured using the typical method in accordance with ASTM D638 (Standard Test Method for Tensile Properties of Plastics), as presented in Table 5. The results of the evaluation of properties revealed that among the DMB samples, DMSPD and TMSPD showed superior performance compared to TESPD, a commercial SCA. The DIN abrasion values of the methoxy-type SCAs—DMSPD and TMSPD—were 129 and 151 mg, respectively, which were smaller than 155 mg of the ethoxy-type SCA—TESPD; similarly, the values of 300% modulus (M_300%_) of the methoxy-type SCAs—DMSPD and TMSPD—were 63.5 and 58.4 kgf/cm^2^, respectively, which were greater than 56.2 kgf/cm^2^ of TESPD. With regard to tensile strength, T-2 obtained by the DMB method using DMSPD also exhibited the highest value (230 kgf/cm^2^). However, there were no significant differences in hardness values. The ethoxy-type DMB sample T-1 showed the highest value in resilience. Therefore, it was possible to conclude that, compared to the ethoxy-type SCAs, the methoxy-type SCAs exhibited the better tire-related mechanical performance. Moreover, SCAs with fewer alkoxy groups (DMSPD) generally demonstrate the best performance in the abrasion resistance, modulus, and the tensile strength [26]. The WMB samples with the methoxy-type SCAs also exhibited the better mechanical properties than the rubber composite sample with the ethoxy-type SCA—T-1. DIN abrasion values of WMB samples were 131 and 137 mg, respectively, and their modulus values were also higher than those of sample T-1. These results may be attributed to the successful optimization of the WMB process with the removal of residual emulsifiers and the sufficient surface modification (i.e., silanization) of the prepared silica cake [25]. In the case of WMB, a much higher production efficiency was also achieved under a mild mixing temperature and a short mixing time as illustrated in the materials and methods section. In particular, the optimized WMB process can introduce the prepared methoxy-type SCAs without any environmental concern, since this technique does not produce the toxic methanol byproduct during the compounding process, as shown in Figure 8. The comparison between WMB samples—T-4 and T-5—also revealed that the rubber composite made from DMSPD, which inherently has fewer alkoxy groups, showed more desirable mechanical properties.

**Figure 9:**
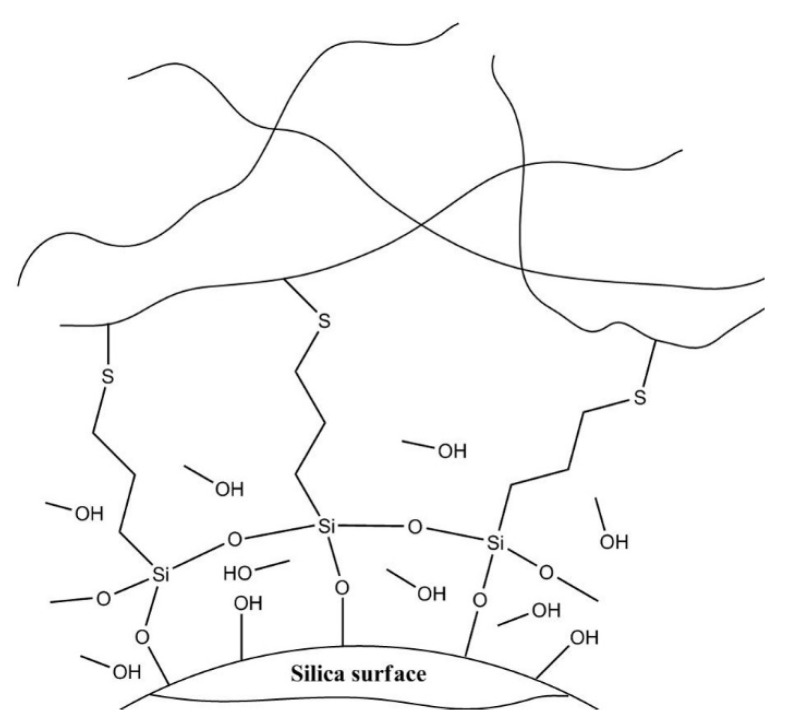
Schematic of the bonding of SCAs to a silica surface and the side reaction of the modified silica.

The reason why the methoxy-type SCAs exhibited superior properties to the ethoxy-type SCAs was that the former had a higher reactivity towards hydrolysis and lower steric hindrance and thus excellent silanization was achieved through the formation of compact bonds with silica. As a result, the effect of silica surface modification was significant, and the higher degree hydrophobicity of silica allowed higher compatibility with SBR. In the comparison between the methoxy-type samples, a reduced number of methoxy groups was more desirable, because fewer methoxy groups bonding with silica led to decreased methanol byproduct generation (see Figure 9), which prevented the weakening of the bond strength between the SBR and the silica surface [25]. Furthermore, as the number of methoxy groups decreased, the effect of steric hindrance became less significant owing to the simple side structure consisting of methyl groups [25]. Therefore, SCAs were be able to bond to the silanol groups of the silica surface more densely despite the smaller number of alkoxy groups [26]. As a result, the effect of the surface treatment on silica became greater, and the hydrophobicity of silica increased to enhance the bond density with SBR, thereby improving various mechanical properties including the abrasion resistance, modulus and the tensile strength. In addition, the hydrophobicity of modified silica increased as a result of fewer polar lateral methoxy groups, which in turn prevented reaggregation among the modified silica particles and avoided adverse effects such as scorch generation and increase in Mooney viscosity.

## 4. Conclusions

In this study, the methoxy-type SCAs—TMSPD and DMSPD—were synthesized and characterized along with the conventional ethoxy-type SCA—TESPD, to investigate the change in properties of silica/SBR composites based on the added SCA structure. The methoxy-type SCAs—TMSPD and DMSPD—showed higher reactivity towards hydrolysis compared to the conventional SCA—TESPD. Therefore, the silica/SBR composites with methoxy-type SCAs were more smoothly fabricated, and the WMB manufacturing method was also possible. Due to the superior silanization resulting from the low steric hindrance of methoxy-type SCAs, the formation of compact bonds with silica was possible, and the various mechanical properties of the composites were improved. In particular, the water-based environmentally friendly WMB process was successfully optimized to introduce these methoxy-type SCAs without any environmental concern. Among the prepared methoxy-type SCAs, DMSPD resulted in more enhanced rubber composite properties than TMSPD, because its fewer methoxy groups led to less methanol byproducts after bonding with silica, which in turn prevented the bond between the SBR and the silica surface from weakening. Moreover, the simplified methyl side group of DMSPD were less affected by steric hindrance and allowed to form more compact bonds with the silica surfaces. The fewer lateral methoxy groups resulted in an increase in the hydrophobicity of the modified silica that suppressed silica reaggregation, thus avoiding undesirable effects such as scorch generation and increase in Mooney viscosity. Consequently, it yielded the best vulcanization characteristics and mechanical properties when manufactured as a rubber compound for tread materials of automobile tire surfaces.

## Figures and Tables

**Table 1:** The mixing compound formulation.

* Phr	T-1 (DMB-TESPD)	T-2 (DMB-DMSPD)	T-3 (DMB-TMSPD)	T-4 (WMB-DMSPD)	T-5 (WMB-TMSPD)
Styrene-butadiene rubber (SBR)-1723	100	100	100	100	100
TDAE oil	37.5	37.5	37.5	37.5	37.5
Dried silica cake	70	70	70		
SCA	SI-75: 7	DMSPD: 7	TMSPD: 7		
DMSPD -modified silica				77	
TMSPD -modified silica				-	77
ZnO	3	3	3	3	3
S/A	2	2	2	2	2
6PPD	1	1	1	1	1
FMB					
Sulfur	1.5	1.5	1.5	1.5	1.5
CBS	1.5	1.5	1.5	1.5	1.5
DPG	1.5	1.5	1.5	1.5	1.5

* Phr: per hundred of rubber.

**Table 2:** The various conditions of hydrolysis reactions.

Sample	SCA (g)	IPA (g)	H_2_O (g)	Acetic acid (g)
1	0.5	4	96	0.7
2		20	80	0.7
3		80	20	0.7
4		96	4	0.7
5		20	80	0
6		80	20	0

**Table 3:** The grafting degree values of the prepared SCAs.

	Pure	MPTMS	DMSPD	TMSPD
R	0.390			
r		0.329	0.283	0.314
K (grafting degree)	0	15.6	27.4	19.5

**Table 4:** The vulcanization reaction characteristics of silica/SBR composites.

	Unit	T-1	T-2	T-3	T-4	T-5
t_10_	min:s	3:36	3:07	2:50	3:34	3:36
t_90_	min:s	6:12	5:17	4:59	5:33	5:55
Cure rate *	N-m/min	0.445	0.528	0.495	0.529	0.513
T_min_	N-m	0.272	0.291	0.341	0.199	0.207
T_max_	N-m	1.260	1.267	1.266	1.266	1.265
T_max_ –T_min_	N-m	0.988	0.976	0.925	1.067	1.058
ML_1+4_	-	98.3	89.9	97.2	86.2	84.5

* (T_60_−T_40_)/(t_60_−t_40_).

**Table 5:** The mechanical properties of silica/SBR composites.

	Unit	T-1	T-2	T-3	T-4	T-5
Hardness	Shore A	57	58	57	57	56
M_100%_	kgf/cm^2^	17.8	19.3	17.1	18.5	17.9
M_300%_	kgf/cm^2^	56.2	63.5	58.4	62.8	60.2
Elongation at break	%	756	692	688	702	736
Tensile strength	kgf/cm^2^	227	230	217	228	223
DIN abrasion	mg	155	129	151	131	137
